# Maintenance of Genome Stability

**DOI:** 10.1016/j.gpb.2016.06.001

**Published:** 2016-06-18

**Authors:** Jiadong Wang, Tomas Lindahl

**Affiliations:** 1 Institute of Systems Biomedicine, Department of Radiation Medicine, School of Basic Medical Sciences, Peking University, Beijing 100191, China; 2 Cancer Research UK London Research Institute, Clare Hall Laboratories, South Mimms EN6 3LD, United Kingdom

It was ever thought that genomic information is transmitted faithfully from generation to generation. But our current knowledge does not indicate that it is the case. For example, genomic variations can be generated from DNA replication infidelity and unequal chromosome segregation. Natural decay of DNA molecules is also a fundamental source of changing genomic information. In addition, cellular and organismal exposure to exogenous genotoxic agents such as ultraviolet (UV) light, oxidative stress, chemical mutagens, and radiation can lead to a variety of modifications on DNA constituents, resulting in genome alterations. Fortunately, cells have evolved several response systems to tackle numerous DNA lesions in order to maintain their genome integrity. Among them, checkpoint control is probably the most well-known one. For example, checkpoint responds to replication stress, replication fork stalling, double-strand DNA breaks, and various other types of DNA lesions. Increasing experimental evidence indicates that genomic instability is probably the fundamental reason for carcinogenesis. Genomic instability is also found to be a main etiological factor of neurodegenerative diseases, aging, immunodeficiency, *etc*. Thus, to understand how cells regulate to maintain their genomic stability is of fundamental importance.

The Nobel Prize in Chemistry 2015 was jointly awarded to Tomas Lindahl, Paul Modrich, and Aziz Sancar for their mechanistic studies of DNA repair. The journal *Genomics, Proteomics & Bioinformatics* (GPB) has thus compiled a special issue Genome Stability following the award announcement. Six mini-reviews are included in this special issue, which cover various aspects of mechanisms underlying the maintenance of genome stability and related diseases. These are designed to introduce our readers to the current understanding, research frontiers, and challenges facing the field.

This special issue starts with a news and views article from Arne Klungland and Yun-Gui Yang [1]. They briefly introduced the main scientific achievements of Tomas Lindahl in unraveling fundamental mechanisms of DNA decay and DNA repair, as well as associations with diseases.

DNA damage is the most important factor that induces genome instability. When DNA repair processes fail, irreparable DNA damage including double-strand breaks (DSBs) can occur. Under normal conditions, DSBs in eukaryotic cells can be repaired by either homologous recombination (HR) or non-homologous end-joining (NHEJ) pathways. A vital step in HR repair is DNA end resection. Liu and Huang [2] reviewed the machinery involved in DNA end resection and described in detail the functions of the most important factors that cooperate to complete the process in eukaryotic cells.

Posttranslational modifications (PTMs) such as phosphorylation, acetylation, methylation, ubiquitination, and SUMOylation are key mechanisms to maintain genome stability. Recently some unique PTMs have been shown to be involved in regulating genome stability. Wei and Yu [3] reviewed the role of poly ADP-ribosylation (PARylation) in DNA repair and genomic stability. PARylation is catalyzed by poly(ADP-ribose) polymerases (PARPs) upon activation by DNA lesions, forming PAR chains that serve as signals and docking platforms for DNA repair factors. These authors [3] highlight the molecular mechanisms of PARylation recognition, the role of PARylation in DDR pathways, and the functional interaction between PARylation and ubiquitination. This review offers a better understanding of the biological roles of this unique PTM in maintaining genome stability.

The review from Dr. Xu’s laboratory [4] is focused on ubiquitin-fold modifier 1 (UFM1), one of the newly-identified PTMs. Similar to ubiquitin, UFM1 is conjugated to its target proteins by a three-step enzymatic reaction, while UFM1 chains are cleaved from target proteins by UFM1-specific proteases (UfSPs), suggesting that the UFMylation modification is reversible. The UFM1 cascade is associated with several cellular activities including the endoplasmic reticulum stress response, hematopoiesis, and certain human diseases. Wei and Xu believe that this reversible modification process might modulate additional cellular activities including tumorigenesis and could serve as potential therapeutic target for cancer treatment.

In addition to protein modifications, post-transcriptional modifications of RNA also have important regulatory roles in cellular processes and might be involved in maintaining genome stability directly or indirectly. Wang and Jia [5] highlighted the functional role of m^6^A reader YTHDC1 in pre-mRNA alternative splicing [6]. Moreover, they also highlighted two transcriptome-wide sequencing methods to identify a new mRNA reversible modification, m^1^A [7], [8]. These two studies provided the first transcriptome-wide methylome map for m^1^A and suggested potential roles for this modification [7], [8]. We expect that further investigation of the wider biological functions of m^6^A and m^1^A, as well as the related writer, eraser, and reader proteins, will build a comprehensive picture of RNA modifications.

Maintenance of tissue-specific stem cells is vital for organ homeostasis and organismal longevity. DNA lesions are direct threats to the genome integrity of the stem cell population. The DDR not only repairs DNA lesions, but also activates orchestrated signaling pathways, leading to cell cycle regulation, cell death and senescence, transcriptional regulation, as well as chromatin remodeling. Recent studies on murine hematopoietic stem cells (HSCs) have indicated that the DDR has important roles in the homeostasis of the hematopoietic system in DDR-deficient mouse models. Li et al. [9] summarized the current understanding of how the DDR intrinsically and extrinsically regulates HSC maintenance, HSC fate determination, and finally organismal aging.

Mutations in certain components of the DDR machinery can lead to genomic instability disorders that culminate in developmental defects, tissue impairment, premature aging, and cancer. Kaminsky et al. [10] reviewed recent progress on the role of the DDR in the etiology of various brain degenerative diseases (BDDs), and summarized the evidence suggesting that BDDs involve the dysfunction of glial cells. Malfunctioning glial cells can severely hamper neural-glial interactions, thereby leading to BDDs.

Li and Liu [11] provided a comprehensive review on the effect of topoisomerase 1 (TOP1) on genome stability. TOP1 relaxes supercoiled DNA to remove helical constraints that can otherwise hinder DNA replication and transcription. Unfortunately, such activity can generate toxic TOP1-DNA covalent products that can lead to cell death or mutagenesis, a precursor for tumorigenesis. In an apparent contradiction to the negative effect of TOP1 activity on genome stability, the detrimental effect of TOP1-induced DNA lesions on cell survival has made this enzyme a prime target for cancer therapies to kill fast-growing cancer cells. The impact of TOP1 research on human health is multifold. In this article, they summarized the current understanding of how TOP1 contributes to human diseases and how its activity can be targeted for disease treatment.

In conclusion, comprehensive understanding of the mechanisms underlying the DDR will not only help us to discover new factors for maintaining genome stability, but also shed light on the development of novel therapeutic strategies for treatment of diseases. We believe that this special issue will serve as a valuable resource to update the current status of genome stability research.

## Competing interests

The authors have declared no competing interests.
